# Outcomes of Different Ablation Approaches for Para-Hisian Accessory Pathway and Ablation Safety at Each Site

**DOI:** 10.3389/fcvm.2021.821988

**Published:** 2022-01-28

**Authors:** Jian-du Yang, Qi Sun, Xiao-gang Guo, Gong-bu Zhou, Xu Liu, Hui-qiang Wei, Hai-yang Xie, Jian Ma

**Affiliations:** ^1^State Key Laboratory of Cardiovascular Disease, Arrhythmia Center, National Center for Cardiovascular Diseases, Fuwai Hospital, Chinese Academy of Medical Sciences and Peking Union Medical College, Beijing, China; ^2^NHC Key Laboratory of Cardiovascular Molecular Biology and Regulatory Peptides, Department of Cardiology, Peking University Third Hospital, Beijing, China; ^3^Department of Cardiology, Beijing An-zhen Hospital, An Affiliate of Capital Medical University, Beijing, China; ^4^Department of Cardiology, Guangdong Cardiovascular Institute, Guangdong Provincial People's Hospital, Guangdong Academy of Medical Sciences, Guangzhou, China

**Keywords:** para-hisian accessory pathway, ablation approach, ablation strategy, atrio-ventricular conduction injury risk, His bundle longitudinal dissociation

## Abstract

**Background:**

This study describes the electrophysiologic characteristics of the para-hisian accessory pathway (AP), the outcome of different ablation approaches, and ablation safety at different sites.

**Method:**

A total of 120 patients diagnosed as para-hisian AP were included in this study. The electrophysiologic characteristics and outcomes at different ablation sites were analyzed.

**Results:**

In total, 107 APs and 13 APs were diagnosed as right anteroseptal (RAS) and right midseptal (RMS), respectively. The significant ECG difference between RAS and RMS was lead III, which mainly manifested as positive and negative delta waves, respectively. Catheter trauma to AP was recorded in 21 of 120 (17.5%) patients. The recurrence rate of direct ablation at the “bumped” sites was higher than the conventional ablation method (37.5 vs. 14.1 %, *p* = 0.036). For RAS APs, there was no significant difference in the success rate between the inferior vena cava (IVC) and superior vena cava (SVC) approaches (76.6 vs. 73.3%, *p* = 0.63). The RAS was separated into three regions: (1) Site 1: superior part above the real “His” recorded site with far-field “His” potential; (2) Site 2 (true para-hisian): the site with near-field “His” potential; and (3) Site 3: inferior part below the biggest real “His” with far-field “His” potential. Mid-septal was defined as an area that is bounded anteriorly by His recording location and posteriorly by the roof of coronary sinus (CS) ostium. The incidence of atrioventricular (AV) conduction injury at different sites was as follows: 3 of 6 (50%) at Site 2, 4 of 13 (30.8%) at RMS, 7 of 34 (20.6%) at Site 3, and 3 of 46 (6.5%) at Site 1. Even if ablation was performed at the atrial side of the para-hisian region, the right bundle branch block (RBBB) was caused in 6 patients (5%).

**Conclusion:**

Ablation *via* IVC or SVC was comparative for para-hisian APs, but not for the noncoronary cusp (NCC) approach. The AV conduction injury risk ranks as follows: Site 2 > RMS > Site 3 > Site 1. RBBB could be caused while ablating at the atrial side, which could further demonstrate the His bundle longitudinal dissociation theory.

## Introduction

Para-hisian atrioventricular (AV) accessory pathways (APs) have been described previously, such as the ECG (1, 2) and electrophysiology characteristics (3) and different ablation strategies (4). However, catheter ablation of the para-hisian AP remains highly challenging (5) because it was easily “bumped” while mapping and highly risky while ablation. Moreover, there was no systematic description of the AV conduction injury risk at different ablation sites and few descriptions of different ablation approaches. In this study, we report a case series of para-hisian APs that successfully ablated by the inferior vena cava (IVC) approach, superior vena cava (SVC) approach, NCC approach, and comparison between different ablation strategies. Otherwise, we will provide risk evaluation at four para-hisian sites.

## Method

### Patient's Characteristics

From 2002 March to 2020 June, a total of 120 consecutive patients diagnosed as APs were presented to our center for invasive electrophysiological evaluation and catheter ablation. Of these, 120 patients with APs arising from the para-Hisian region were included in this series. In total, 105 patients (87.5%) were documented as having frequent symptomatic episodes of narrow QRS complex tachycardia. There were 82 (68.3%) men and 38 (31.7%) women, with a mean age of 33 ± 16 years (range 7–64 years). All patients gave written informed consent before the procedure. The research protocol used in this study was reviewed and approved by the institutional review board of Fuwai Hospital.

### ECG and EP Data Analysis

ECGs during sinus rhythm and during maximal pre-excitation were independently evaluated by 2 observers, and the delta-wave morphology was described as positive, negative depending on the deviation from baseline. Any discrepancy was resolved by consensus.

### Baseline Electrophysiological Study

After providing written informed consent, patients underwent invasive electrophysiological testing. Femoral venous access was obtained and one quadripolar catheter was advanced to the right ventricular apex (RVA), another quadripolar/hexapolar catheter to His bundle area, and a decapolar catheter was placed in the coronary sinus (CS) *via* the right internal jugular vein. Data were recorded simultaneously by a digital multichannel system (LabSystem PRO, Bard Electrophysiology, Lowell, MA, USA). Bipolar signals were filtered at 30–500 Hz, and unipolar signals were filtered at 0.05–500 Hz.

### Mapping and Ablation

After the presence of retrograde APs or manifest APs were suspected to be para-hisian originated according to existing criterion (6–8). The mapping and ablation protocol was performed as follows: the earliest activation region in the right atrium/ventricle was initially mapped during antegrade/retrograde AP conduction *via* the femoral vein. If the catheter through the IVC approach was not stable, the SVC method or Swartz long sheath *via* IVC would be tried. When the APs could not be terminated from the method above, mapping and ablation through NCC was attempted. Aortic angiography was performed in all patients before catheter ablation to determine the location of the coronary arteries and delineate the anatomy of the coronary cusps.

Among these patients, a three-dimensional electroanatomic mapping system (CARTO, Biosense Webster, Inc., Diamond Bar, CA, USA) was used in 64 patients. Radiofrequency energy delivery was started at relatively low power (10–15 W) and then gradually titrated up to 20–40 W according to the AP location (right anteroseptal [RAS] could reach 40 W, right midseptal [RMS] was up to 25 W) using a 4-mm non-irrigated ablation catheter (ABL). The endpoint of the procedure was a loss of pre-excitation and no AP retrograde conduction.

### Follow-Up

After RF catheter ablation, patients were monitored for 24–48 h with inpatient telemetry, standard 12-lead ECGs, and a 24 h Holter. The first post-discharge follow-up appointment was arranged 1 month after ablation in some patients. Telephone interviews were performed with patients or family members to confirm the absence of the arrhythmia symptoms and pre-excitation on 12-lead ECGs.

### Definition

#### Near-Field “His” Potential and Far-Field “His” Potential

It was mainly differentiated by two methods: (1) The information derived from the amplitude with the information derived from the slope. According to the geometrical relationship between wavefront and electrode with a wave that is far–from vs. near–to the recording site. A far-field signal moves toward and then recedes from the electrode without ever getting close, and therefore such a signal never gets to a proximity where its slope is steep; i.e., far-field signals are more rounded than near-field. Therefore, a “sharp” with/without large amplitude “His” electrogram is preferred to be near-field potential. Otherwise, it was preferred to be far-field “His” potential. (2) The pacing maneuver described by Xue et al. (9), the right-sided near-field “His” was defined as the presence of His capture with minimal pacing output. Far-field “His” sites were defined if they required >10 mA/2 ms to capture the His bundle.

**Figure 1:**
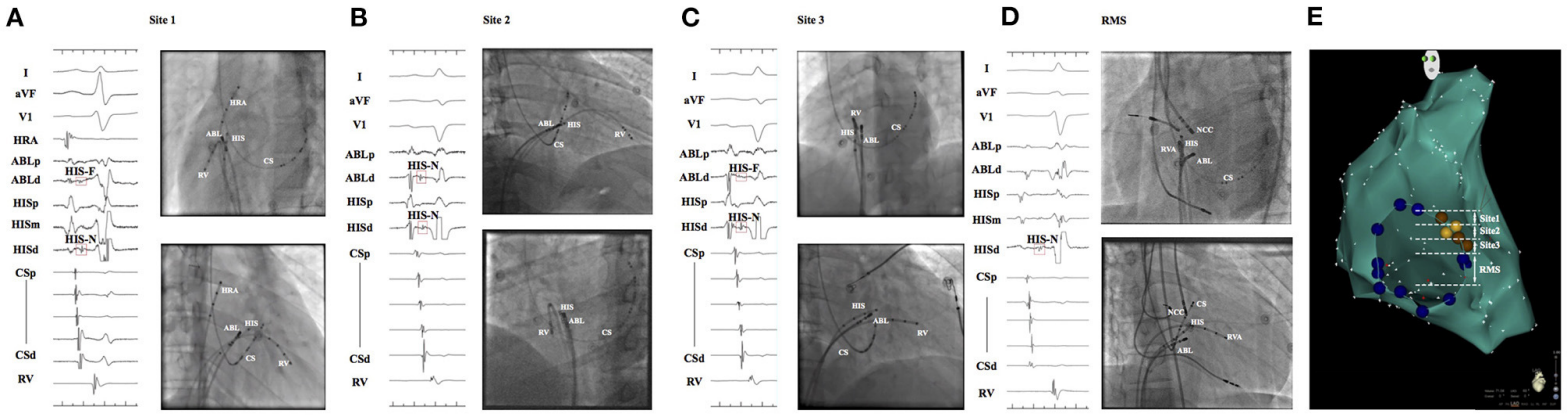
**(A)**, ABL was at site 1, which locates at a superior part above the real “His” with far-field “His” potential recorded; **(B)**, Site 2 (true para-hisian): the site with near-field “His” potential; **(C)**, Site 3: inferior part below site 2 with far-field “His” potential recorded; **(D)**, RMS: the site bounded anteriorly by His recording location and posteriorly by the roof of CS ostium without “His” potential recorded; **(E)**, left anterior oblique view of the tricuspid annulus and right anterior septum was divided into three subgroups. Real “His” was labeled with yellow dots and far-field “His” was marked with brown dots. ABL, ablation catheter; RMS, right midseptal.

**Figure 2:**
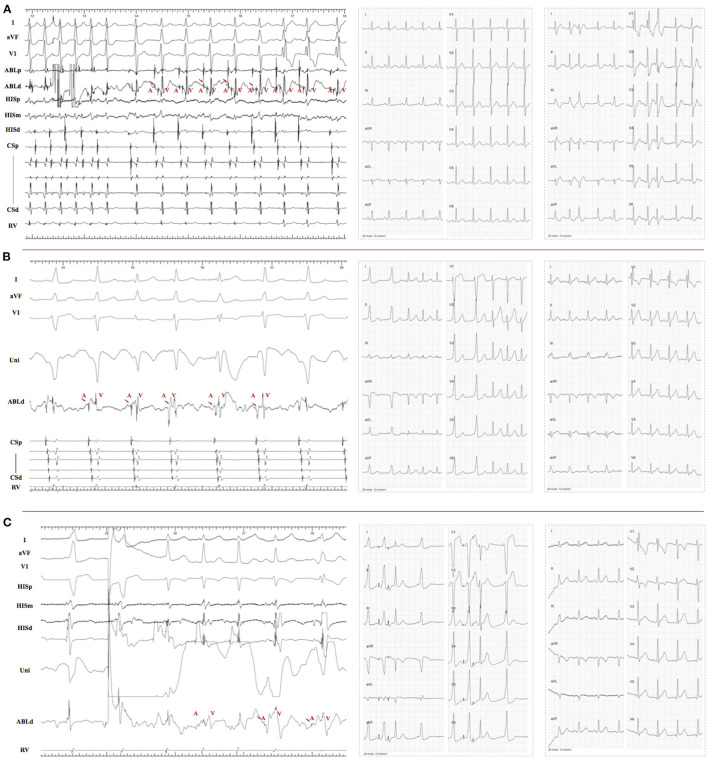
**(A)**, left panel, RF performed at site 2 and RBBB obtained during ablation. There was “A,” “H,” and “V” recorded at the ablation target; middle panel, ECG before ablation; right panel, right bundle conduction recovered after RF off. **(B)**, left panel, RF performed at the atrial side of site 3 and caused RBBB during ablation; middle panel, narrow QRS tachycardia without RBBB induced before ablation; right panel, RBBB still existed at the end of the procedure. **(C)**, left panel, RF performed at site 1 with small “A” potential and lead to RBBB; middle panel, narrow QRS observed during A-pacing before ablation (third beat); right panel, RB conduction recovered when RF terminated. RBB, right bundle branch.

Anteroseptal APs were considered to be located in the apex of Koch's triangle at a site from which a small His potential can usually be recorded (10). It was divided into three accurate subgroups: (1) Site 1: superior part above the real “His” recorded site with far-field “His” potential (Figure 1A); (2) Site 2 (true para-hisian): the site with near-field “His” potential (Figure 1B); and (3) Site 3: inferior part below the biggest real “His” with far-field “His” potential (Figure 1C).

Mid-septal APs are defined if the APs could be ablated successfully in an area that is bounded anteriorly by His recording location and posteriorly by the roof of CS ostium (Figure 1D).

Conduction injury was defined as temporary/permanent AV block that includes complete AV block or PR prolongation, right bundle branch block (RBBB).

### Statistical Analysis

Values are given as mean ± SD. All the continuous parameters were assessed by Student's t study or the Kruskal-Wallis non-parametric test, and all categorical variables were expressed as percentages and analyzed by the Chi-sequence test or Fisher's exact test. *p* < 0.05 was considered significant differences. SPSS, version 22.0 (SPSS, Inc., Chicago, IL, USA) was used for statistical analysis.

## Results

### Patient Characteristics

Of the 120 patients, 107 APs and 13 APs were regarded as anteroseptal and midseptal, respectively. Excluded 21 patients from anteroseptum without X-ray or Carto data who could not find out the specific ablation site, 47 APs were eliminated from site 1, 5 APs were eliminated from site 2, and the left 34 APs were from site 3.

### ECG Characteristics

#### Anteroseptal APs

In anteroseptal APs, 50 of 107 patients (46.7%) exhibited manifest ventricular pre-excitation on their baseline 12 leads ECG and 12 of them had only antegrade conduction. All patients with ventricular pre-excitation showed a positive delta wave polarity in leads I and II, and negative in lead aVR; most of the patients showed positive delta wave in leads III (49 of 50, 98%), aVL (38 of 50, 76%), aVF (44 of 50, 88%), and negative delta wave in lead V1 (38 of 50, 76%). Precordial transition varies: V2 transition was recorded in 20 patients (40%), V2–V3 transition in 13 patients (26%), and >V3 transition in 17 patients (34%).

### Mid-Septal APs

Nine of 13 patients (69.2%) exhibited manifest ventricular pre-excitation and 2 patients had only antegrade conduction. Similar to anteroseptal APs, all the patients had a positive delta wave in leads I and II, negative in lead aVR; the majority of the patients could record negative delta wave in leads III (7 of 9, 77.8%) and V1 (6 of 9, 66.7%), positive delta wave in leads aVL (8 of 9, 88.9%), and aVF (5 of 9, 55.6%). Precordial transition varies: V2 transition was recorded in 4 patients (44.4%), V2-V3 transition in 3 patients (33.3%), and >V3 transition in 2 patients (22.2%).

### Mechanical Trauma to Pathways

Catheter-trauma to the AP is very common during catheter manipulation. In this study, 21 of 120 (17.5%) patients could record AP block during mapping. There was no significant difference in age (31 ± 14 y vs. 33 ± 16 y, *p* = 0.57), gender (*p* = 0.43), prior ablation history (28.6% vs. 11.1%, *p* = 0.0768), and AP location (18.7% of RAS vs. 7.7% of RMS, *p* = 0.46) of patients with and without catheter-induced trauma. However, the APs involved were manifest in 19 (90.5%) patients, concealed in 2 (9.5%), which correlated with the occurrence of trauma (*p* = 0.0004). Two APs (9.5%) could be blocked with His catheter, and all 21 patients obtained mechanical trauma during mapping with the ABL. The median recovery time was 29 s (range from 5 to 21 min) after bump block. All of the patients performed ablation at the “bumped” sites, but the recurrence rate was relatively higher which could reach 37.5% (6 of 16, 5 patients lost follow-up) compared with the recurrence of 14.1% (12 of 85, 14 patients lost follow-up) with the conventional approach (*p* = 0.036).

### AV Conduction Injury

Exclude one patient in the RMS group who did not perform ablation because mechanical trauma-induced AV block was observed during mapping, the overall incidence of RF caused AV conduction injury was 15.9% (17 of 107 patients) and 33.3% (4 of 12 patients) in the RAS and RMS groups, respectively. Since the imaging data were not available for 21 patients in the RAS group and specific locations cannot be determined, these 21 patients were excluded. The incidence of RF caused conduction injury of the specific site was as follows: 3 of 6 (50%) at Site 2, 4 of 12 (30.8%) at RMS, 7 of 34 (20.6%) at Site 3, and 3 of 46 (6.5%) at Site 1. Moreover, the injury type was quite different between groups. RBBB was well documented (2 of 3, 66.7%) during ablation at site 1. Otherwise, it was easier to get temporary/permanent AV block below this site, i.e., 2 of 3 (66.7%) at Site 2, 3 of 4 (75%) at RMS (i.e., one patient who had temporary PR prolongation and permanent RBBB), and 6 of 7(85.7%) at Site 3. More remarkably, there were 4 patients who got temporary (3) or permanent (1) III° AVB during ablation, all occurred during ablation at Site 3 (2) and RMS (2). RBBB was observed in 6 patients, which happened during ablation at the atrial side (Figure 2).

### Different Ablation Approach and Outcome

**Figure 3:**
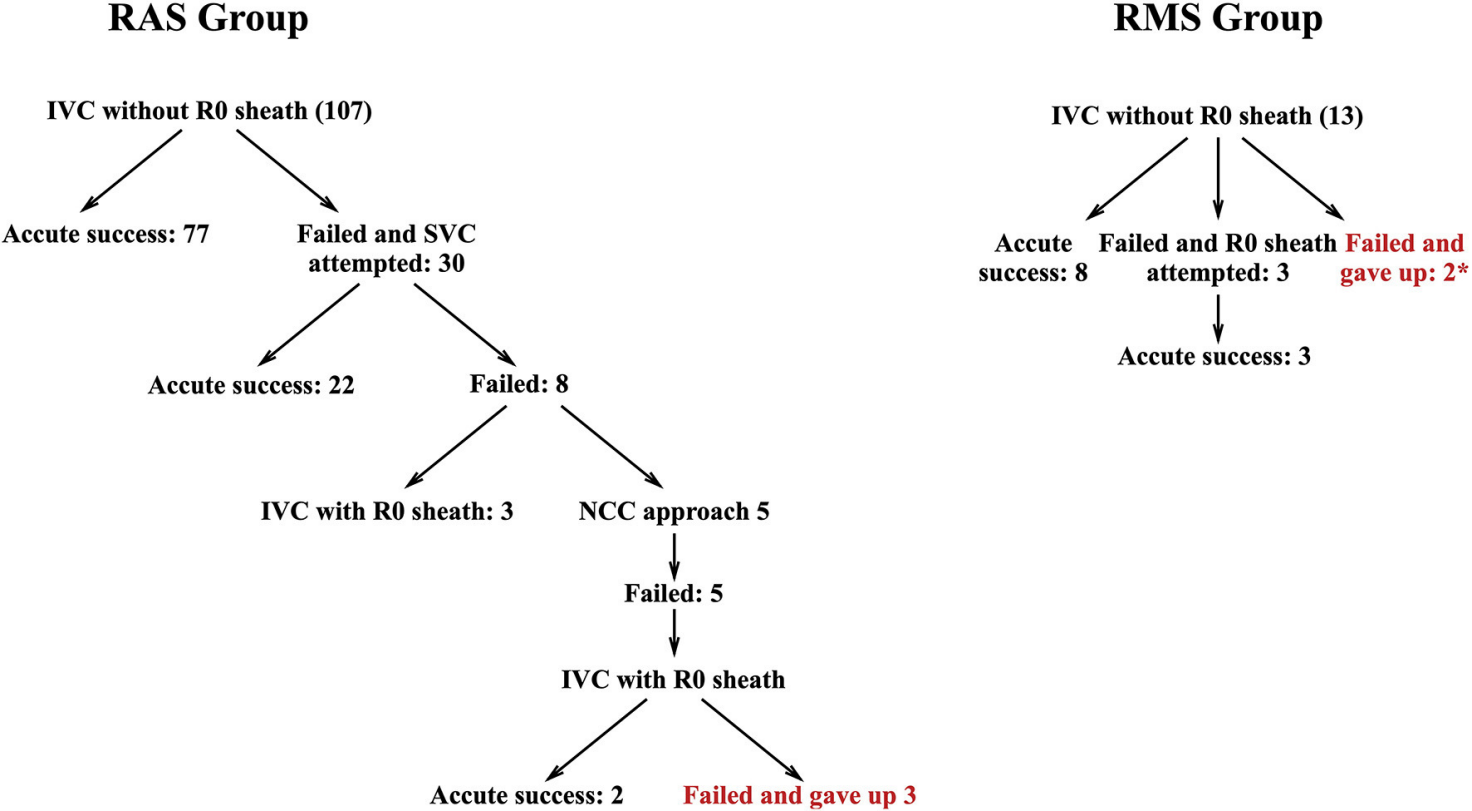
Flowchart of ablation protocol in RAS and RMS group.* 1 patient gave up directly because of mechanical trauma-induced AV block and another one had AV block during ablation. RAS, right anteroseptal; RMS, right midseptal.

Except 3 APs from RAS and 2 APs from RMS gave up because of the high risk of AV block, the remaining APs were successfully eliminated during ablation. In the RAS group, successful ablation through the IVC approach was achieved in 82 patients (76.6%), such as 5 (4.7%) with Swartz R0 sheath. The remaining 22 (20.6%) APs were eliminated through the SVC approach. For RAS APs, there was no significant difference in success rate between the IVC and SVC approaches (76.6 vs. 73.3%, *p* = 0.63). However, 11 of 13 (84.6%) patients in the RMS group reach successful ablation through the IVC approach, i.e., 3 (23.1%) with R0 sheath (Figure 3). Five patients in the RAS group attempted the NCC approach, but all failed. Because of the distance between NCC and RMS, the NCC approach was not attempted in the RMS group.

### Follow-Up

Nineteen patients were lost during an average follow-up of 65 ± 50 months, such as 17 in the RAS group and 2 patients in the RMS group. Sixteen patients recurred in the RAS group (including 3 failed ablation) and 2 patients with failed ablation recurred in the RMS group.

## Discussion

### ECG Characteristics and Prior Investigations

ECG characteristics of anteroseptal and midseptal were described in several studies. In patients with anteroseptal AP, Rodriquez et al. (2) reported the presence of positive delta waves in all inferior leads.

Xie et al. (1) reported the positive QRS complex in lead aVF and the negative complex in lead III in patients with anteroseptal AP. Haghjoo et al. (11) reported a positive delta wave in leads I, II, III, aVL, aVF, and V2–V6 with a precordial transition zone in leads V3 and V4. Otherwise, in our study, a positive delta wave was shown in leads I, II, and most of the leads III and aVF, but isoelectric/negative delta wave could also be recorded in lead aVF (12%) in some of the patients. The precordial transition was various: V2 transition was recorded in 20 patients (40%), V2–V3 transition in 13 patients (26%), and >V3 transition in 17 patients (34%).

In patients with midseptal APs, the main difference from anteroseptal APs was the inferior leads. Kuck et al. (12) showed the presence of predominantly negative delta waves in leads III and aVF. Tai et al. (3) reported a negative delta wave in lead III and isoelectric delta wave in aVF. Except for this ECG characteristic, Yang et al. (10) also reported that precordial transition occurred between leads V1 and V2. However, in our study, although a majority of the patients could record negative delta wave in lead III (7 of 9, 77.8%), positive delta wave could also be recorded in the remaining two patients, and the precordial transition was also various and no difference with anteroseptal APs.

### The Proper Way to Deal With the Catheter Trauma

Although ablation at the “bumped” site was considered as an alternative method for AP, it was not a satisfactory method with a high recurrence rate (30%). In our study, the spontaneous conduction recovery time was quite short after the mechanical block happened. Therefore, the wise choice was to withdraw the offending catheter immediately and repeat mapping after the pathway conduction resumes.

### Prior Investigations and Different Ablation Approaches

As we all know, the IVC approach was the most common way for septal AP ablation. After Tada et al. (13) first reported a case that an anteroseptal AP was successfully eliminated from NCC, this approach has been suggested as an alternative approach for this particular AP (14, 15). Besides, DiLorenzo et al. (16) described another technique of ablation of anteroseptal APs *via* the right internal jugular, it provided another ablation method for anteroseptal AP. Recently, Liang et al. (4) reported that most of the cases could be successfully ablated by the IVC approach, only 4 of 55 (7%) and 3 of 55 (6%) para-hisian APs require NCC approach and SVC approach, respectively. In their study, they first provided the constituent ratio of the para-hisian APs ablation method. Different from previous studies, except for the patients with acute failed ablation (3 of 107, 2.8%), there were 22 (20.5%) patients who had successful ablation *via* SVC and further proved this was a significant approach for this particular group. Moreover, different from Xu et al. report (17) that radiofrequency delivered at the NCC had a higher success rate (11 of 12, 92.7%) vs. right anterior septum (5 of 12, 41.7%). In our study, the NCC approach was attempted in 5 patients after failed ablation through IVC and SVC approaches, but none of them succeed.

### Anatomic Consideration and Relationship Between Ablation Site and AV Conduction System Injury Type

**Figure 4:**
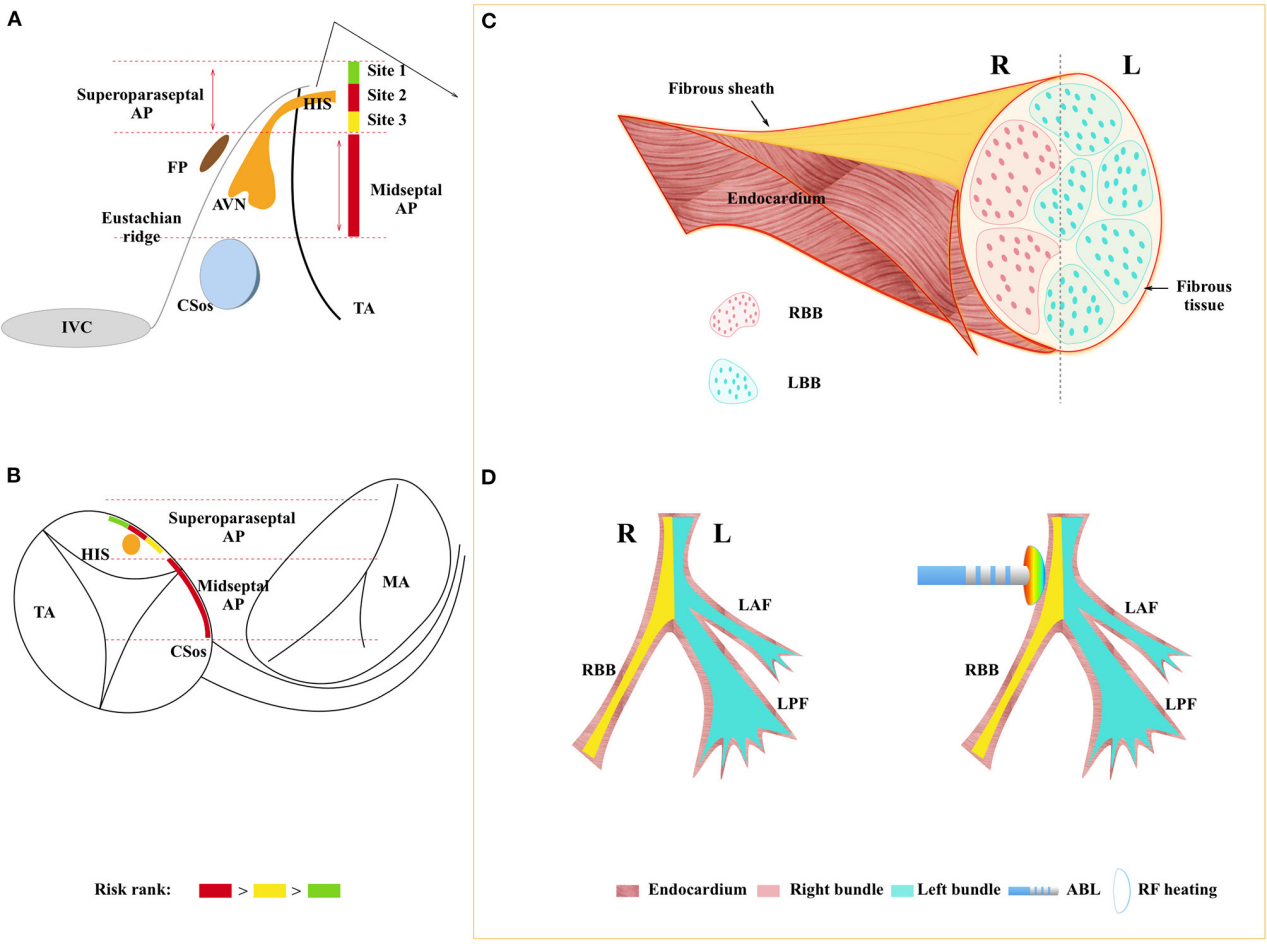
**(A)**: Schematic representation of the septal region. **(A)**: right anterior oblique (RAO) view. **(B)**: left anterior oblique (LAO) view. The septal region usually is bounded superiorly by the His bundle (HBE) and inferiorly by the roof of the coronary sinus (CS). Dividing this area into three parts, para-Hisian accessory pathways occupy the superior third of the area and midseptal APs occupy the middle third. Site 2 and midseptal are the most risky sites, following with site 3 and site 1. **(C)**: His-Purkinje anatomy with longitudinal dissociation and it was surrounded by a fibrous sheath, right bundle branch, and the left bundle branch close to each side of the endocardium. **(D)**: left panel shows the overall view of the His-Purkinje system; the right panel shows the RF delivered at the para-hisian region, heat could transfer from the endocardium to the right bundle inside which could cause RBBB as consequence. AVN, AV node; IVC, inferior vena cava; TA, tricuspid annulus; MA, mitral annulus; HIS, His bundle; AP, accessory pathway; FP, fast pathway; RBB, right bundle branch; LBB, left bundle branch; LAF, left anterior fascicle; LPF, left posterior fascicle; ABL, ablation catheter; Endo, Endocardium.

Koch's triangle is delineated by the Eustachian ridge, the membranous septum, and the insertion of the tricuspid valve. The atrioventricular node is located at the right mid-septum and its anterosuperior extension, the His bundle, penetrates into the fibrous body through the apex of the Koch's triangle to become surrounded by fibrous tissue. However, the nodal body adjoins the atrial aspect and making it an interatrial structure. In the adult heart, the minimal distance of the compact node from the endocardium is about 0.5–1.5 mm (18). Moreover, there is no fibrous tissue around the AV node which makes it a highly vulnerable tissue compared with His bundle. In a word, His bundle is located more superior than the AV node and is relatively more resistant to radiofrequency because of the surrounded fibrous sheath. The anatomical characteristics of the conduction system could explain that the incidence of conduction injury was less documented at site 1 (6.5%), but relatively more at lower sites. Hence, according to the incidence of conduction injury and different injury types, we ranked the ablation risk of the following four sites: Site 2 > RMS > Site 3 > Site 1 (Figures 4A,B).

It was well known that the His bundle bifurcates into the left and right bundles. In the 1970's, James and Sherf first reported that there are longitudinally oriented cells separated by collagen sheaths (19) within the His bundle. According to this, they posited that the His bundle was longitudinal dissociation. To date, the His bundle pacing was proved to be useful in patients with complete left bundle branch block (20). More and more evidence proved the theory of His bundle longitudinal dissociation. In the previous study (21), it was considered that the right bundle block was caused when the catheter was positioned too distally and the right bundle was targeted. In our study, RBBB was obtained during ablation among 6 patients, all of these happened during ablation at the atrial side of tricuspid annulus (TA) (Figure 2). It could only be explained by the longitudinal dissociation theory, and the conduction system is sandwiched between the right and left endocardium (Figures 4C,D). In other words, right bundle branch (RB) and left bundle branch (LB) were close to each side of the endocardium, respectively. Hence, if radiofrequency was delivered at the right atrium endocardium, the energy would transfer deep inside and cause injury to the right bundle as a consequence.

## Limitation

This is a single-center, retrospective study, and the ablation approach was chosen according to the physician's preference. In addition, cryoablation technology was not available for use at our institution during the study period. Moreover, the duration of the study is 18 years during which the techniques and the operators might have changed. This may have a significant impact on the outcomes.

## Conclusion

Ablation *via* IVC or SVC was comparative for para-hisian APs, but not with the NCC approach. The AV conduction injury risk ranks as follows: Site 2 > RMS > Site 3 > Site 1 and close attention should be paid while ablating is at Site 2 and RMS. RBBB could be caused even if ablation is performed at the atrial side of the para-hisian region, which could further demonstrate the His bundle longitudinal dissociation theory.

## Data Availability Statement

The original contributions presented in the study are included in the article/supplementary material, further inquiries can be directed to the corresponding author/s.

## Ethics Statement

The studies involving human participants were reviewed and approved by Institutional Review Board of Fuwai Hospital. The patients/participants provided their written informed consent to participate in this study.

## Author Contributions

J-dY, QS, and JM conceived the study. J-dY and X-gG collected the data. H-yX analyzed the data. G-bZ, XL, and H-qW critically revised the article. All authors contributed to the article and approved the submitted version.

## Funding

This study was supported by a grant from the National Natural Science Foundation of China (#81670309).

## Conflict of Interest

The authors declare that the research was conducted in the absence of any commercial or financial relationships that could be construed as a potential conflict of interest.

## Publisher's Note

All claims expressed in this article are solely those of the authors and do not necessarily represent those of their affiliated organizations, or those of the publisher, the editors and the reviewers. Any product that may be evaluated in this article, or claim that may be made by its manufacturer, is not guaranteed or endorsed by the publisher.
